# Positive impact of a telemedicine education program on practicing health care workers during the COVID-19 pandemic in Ontario, Canada: A mixed methods study of an Extension for Community Healthcare Outcomes (ECHO) program

**DOI:** 10.1177/1357633X211059688

**Published:** 2021-12-28

**Authors:** Q. Jane Zhao, Dmitry Rozenberg, Sahar Nourouzpour, Ani Orchanian-Cheff, John Flannery, Rupert Kaul, Senyo Agbeyaka, Mary Barber, Patrice dePeiza, Anna Maria Doumouras, Haley Draper, Nadine Gebara, Jenny Lau, Dan Liberman, Ryan A Luther, Monica Sanh, Andrea D Furlan

**Affiliations:** 1 ECHO at UHN, University Health Network, Toronto, Canada; 2 Mount Sinai Hospital, 12367 Toronto, Canada; 3 Temerty Faculty of Medicine, University of Toronto, Toronto, Canda; 4 Library and Information Services, University Health Network, Toronto, Canada; 5 Department of Family and Community Medicine, University of Toronto, Toronto, Canada; 6 Department of Medicine, McGill University; 7 Jewish General Hospital, Montreal, Quebec, Canada

**Keywords:** telemedicine, COVID-19, primary care, mixed methods, continuing medical education, ECHO (Extension for Community Healthcare Outcomes)

## Abstract

**Introduction:**

In addition to shifting and expanding clinical responsibilities, rapidly evolving information and guidelines during the COVID-19 pandemic has made it difficult for health care workers (HCW) to synthesise and translate COVID-19 information into practice. This study evaluated whether a COVID-19-specific telemedicine education program (ECHO COVID) would impact health care workers’ self-efficacy and satisfaction in the management of patients with COVID-19.

**Methods:**

A prospective mixed methods parallel-design study was conducted among ECHO COVID participants using pre-post questionnaires and a focus group discussion. Questionnaire results were examined for changes in health care workers’ self-efficacy and satisfaction. Focus group discussion data were analysed to explore health care workers’ experience in ECHO COVID and the context of their practice during the COVID-19 pandemic.

**Results:**

239 health care workers registered in ECHO COVID and 114 (47.7%) completed questionnaires and attended at least one ECHO COVID session. Median self-efficacy scores increased from 5 (IQR 4–6) to 6 (IQR 6–6) (*p* < 0.0001), independent of profession, years in practice, age group, or practice environment. Participants were highly satisfied with ECHO COVID sessions with a median score of 4 (IQR 4–5). Focus group discussion data indicated that health care workers gained knowledge through ECHO COVID and revealed facilitators for ECHO COVID program success, including the transition to virtual care, the practicability of knowledge provided, and a ‘perspective from the trenches.’

**Discussion:**

This study demonstrated that a telemedicine education program aimed to support health care workers in managing patients with COVID-19 had a positive impact on health care workers’ self-efficacy and satisfaction. This impact was specifically mediated by the ECHO COVID program.

## Introduction

The impact of the COVID-19 pandemic on health care service and delivery has been profound.^1–4^ In addition to navigating the transition to virtual care,^5–7^ staffing and clinic changes,^8–12^ and vaccine distribution challenges,^13^ health care workers (HCWs) have had to learn about the novel SARS-CoV-2 virus. Many HCWs worldwide are experiencing increased psychological distress^14–18^ and burnout.^19–21^ In Canada, the burden of COVID-19 has put strain on the health care system.^22–25^ Furthermore, HCWs have difficulty synthesising and applying evidence-based COVID-19 guidelines and research into practice due to the rapidly evolving changes in guidelines, volume, and quality of information.^26–28^

Project **E**xtension for **C**ommunity **H**ealthcare **O**utcomes (ECHO) model is a virtual, telemedicine education model that provides longitudinal support and addresses the emerging needs of HCWs.^29^ ECHO programs have been successfully implemented internationally, providing rapid access to evidence-based research, policy, and practice information.^30–33^ In April 2020, University Health Network (UHN) leveraged the pre-existing ECHO model as a model of care to support assessment and management of COVID-19 patients. ‘ECHO Ontario: Managing COVID-19 Patients in the Community’ (ECHO for the remainder of this paper) was launched in July 2020. The goal of the program was to disseminate best practices regarding COVID-19 as they emerged and to increase HCWs’ self-efficacy and knowledge of COVID-19.

The objectives of this study are: (1) to examine the impact of a telementoring education program on HCWs’ self-efficacy and satisfaction and (2) to explore HCWs’ experience in the program and context of practice during the COVID-19 pandemic.

## Methods

### Study setting

The ‘ECHO Ontario: Managing COVID-19 Patients in the Community’ program was represented by an interprofessional ‘hub’ team, including medicine (family medicine, infectious diseases, internal medicine, respirology, critical care, palliative care, and physiatry), occupational therapy, naturopathy, pharmacy, physiotherapy, social work, and information specialists. The target audience was any practicing HCWs in Ontario.

Each ECHO session ran on Tuesdays for one hour. Weekly sessions included a didactic lecture and a patient presentation.^34^ All sessions were recorded and available for review after sessions. Participants attended at no-cost and received Continuing Medical Education (CME) or Continuing Professional Development credits for their attendance.

A needs assessment was conducted to develop the didactic curriculum, based on available resources from the international ECHO (MetaECHO) community.^35^ The final curricula included: classification of COVID-19, pharmacotherapy, management of special populations, community resources, respiratory health, etc. (Appendix 1). Patient lived experiences as didactic sessions were also incorporated in each cycle, which offered a unique perspective on care.

### Study design

A prospective mixed methods parallel-design study was conducted to evaluate the impact of ECHO on HCWs.^36^ A pre-post questionnaire was administered through Survey Monkey®. A focus group discussion (FGD) was conducted to explore the context of practice during the COVID-19 pandemic and the impact of ECHO on their practice. Data were collected from both parts of the study concurrently and analysed.

### Study participants

Participants were recruited from June 2020 to March 2021. Participant eligibility for this study included (1) all HCWs (family medicine and specialist physicians, physician assistants, nurse practitioners, registered nurses, registered practical nurses, pharmacists, and other allied health professionals), (2) practicing in Ontario and (3) attended at least one ECHO session. Exclusion criteria included all HCWs practicing outside of Ontario, Canada or those not actively in practice.

### Data sources: Pre-post questionnaires

The Pre-Post questionnaires were self-reported and contained sections on self-efficacy and acceptability and satisfaction. Demographics and practice characteristics were collected.

**Self-efficacy** is defined as an individuals’ confidence in their ability to perform a particular task.^37^ In addition to knowledge, self-efficacy has been demonstrated to be an important mediator for behavioural change among practicing HCWs.^38,39^ Participants reported self-efficacy using a 17-item scale, adapted from previous ECHO programs. Each item in this section was assessed on a 7-point Likert scale which ranged from 1 = ‘strongly disagree’, to 7 = ‘strongly agree’. Responses of ‘not applicable’ for any item were excluded. Self-efficacy items included ability to diagnose COVID-19, to identify which tests to order, and to manage special patient populations.

**Acceptability and satisfaction with ECHO** was measured using an 11-item scale. This section used a 5-point Likert scale where 1 = ‘strongly disagree’ and 5 = ‘strongly agree’, with a ‘not applicable’ option. Items included statements like ‘I believe that the quality of care I provide my patient has improved considerably as a result of my involvement in project ECHO’ and ‘Project ECHO has reduced variations in care’.

### Data sources: focus group discussion

One FGD was conducted in November 2020 with participants from the first cycle. The group consisted of five study participants with one facilitator (QJZ) and one note taker. The FGD was conducted and recorded over Zoom. The recording was transcribed verbatim.

Purposive sampling was used to recruit participants who had completed the pre-ECHO questionnaire. This sampling strategy was employed in order to represent a broad cross section of participants, including professions, practice types, and years in practice.

The FGD guide was developed with the research team, containing open-ended questions (Appendix 2). Two broad topics of inquiry included 1) HCWs’ experience in ECHO and the impact on their practice, and 2) the context of practice during the COVID-19 pandemic. All FGD participants received a $50 honorarium for their time.

### Data analysis

#### Statistical analysis

Statistical analyses were performed using GraphPad Prism 9.0 (GraphPad Software, Inc., San Diego, CA). For participant characteristics, self-efficacy, and satisfaction responses, continuous and categorical variables were used including mean ± standard deviation, median (interquartile range [IQR]), frequencies (n), and percentages, as applicable. Data distribution was evaluated visually and with the use of Kolmogorov-Smirnov and Pearson omnibus normality tests. Statistical significance between groups was assessed using t-tests (parametric and non-parametric) and chi-square for categorical variables. The changes in self-efficacy scores were evaluated using paired t-tests. Those with missing data for a particular parameter or question were excluded. A *p*-value of <0.05 was considered significant for all analyses.

#### Qualitative data analysis

The qualitative descriptive method was employed to synthesise and analyse the focus group transcript. This analysis method was used because it allowed us to produce a comprehensive summary of the data in plain language and to stay close with the original meaning of the data.^40,41^ Two members of the study team (QJZ, SN) reviewed the questionnaire data and transcript to develop a coding framework of recurrent themes. The themes were then presented to a small working group (DR, SN, AF). Through iterative discussion during the analysis phase, all themes were discussed and discrepancies were resolved verbally.

Qualitative content analysis was conducted inductively to identify emergent themes from the data and develop a framework for coding.^42,43^ Codes identified the topics in the data pertinent to the research objectives. Data were then grouped into broader themes. All qualitative data was analysed using NVivo (NVivo 11 for Windows, Version 11.0.0.300 by QSR International).

### Ethics approval

This study received an exemption by the UHN Research Ethics Board and was approved through the UHN Quality Improvement stream (QI ID#20-0039).

## Results

Between July 2020 and February 2021, ECHO offered 24 sessions (twelve per cycle). 239 HCWs registered for the program and of those, 114 (47.7%) attended at least one ECHO session and completed at least one of the pre-post questionnaires. 50 (43.9%) completed both pre-post questionnaires and were grouped for subgroup analyses. Completion of the pre-ECHO questionnaire took 4 min and 19 s on average, while completion of the post-ECHO questionnaire took an average time of 4 min and 38 s. Comparing HCWs who registered but did not attend any sessions and HCWs who registered and attended at least one ECHO session with completion of questionnaires, differences were observed between groups for years in practice (*p* = 0.0009), age group (*p* = 0.0006), and number of patients with COVID-19 (*p* = 0.0023) (Appendix 3).

### Participant characteristics

Of the 114 HCWs who were included in this study, 99 participants were female (86.8%), 42 identified as a nurse practitioner (36.8%), and nearly half reported to be in their first ten years of practice (49, 43.0%) (Table 1). 42 ECHO participants reported practicing in a family health team (36.8%), while 82 worked in an urban setting (71.9%), and 45 (39.5%) reported no patients with COVID-19 in their practice. Participants in the total cohort had a median attendance of 3 ECHO sessions (IQR 2–6). The participants in the total cohort attended fewer sessions than those in the subgroup with a median of 3 [2–6] and 4 [3–9] sessions, respectively (*p* = 0.014). No other statistical differences between participant characteristics in the total cohort and subgroup were observed.

**Table 1.** Baseline cohort characteristics of participants.

	Total cohort (*n* = 114)	Subgroup^a^ (*n* = 50)	*p*-value
**Profession**			0.94
NP	42 (36.8%)	26 (52.0%)	
MD	34 (29.8%)	14 (28.0%)	
Other	38 (33.3%)	10 (20.0%)	
**Female**	99 (86.8%)	44 (88%)	0.84
**Years in practice**			0.70
0–9	49 (43.0%)	21 (42.0%)	
10–19	30 (26.3%)	14 (28.0%)	
≥20	21 (18.4%)	13 (26.0%)	
Unknown	14 (12.2%)	2 (4.0%)	
**Age group**			0.51
20–39	47 (41.2%)	18 (36.0%)	
40–59	40 (35.1%)	20 (40.0%)	
≥60	15 (13.2%)	10 (20.0%)	
Unknown	12 (10.5%)	2 (4.0%)	
**Type of practice** ^b^			0.95
Family health team	42 (36.8%)	20 (40.0%)	
Hospital	19 (16.7%)	10 (20.0%)	
Independent practice	13 (11.4%)	6 (12.0%)	
Community health center	10 (8.8%)	7 (14.0%)	
Long-term care	8 (7.0%)	4 (8.0%)	
Family health organization	6 (5.3%)	4 (8.0%)	
Other	16 (14.0%)	13 (26.0%)	
Two or more groups selected	11 (9.6%)	4 (8.0%)	
**Attendance**			0.17
Attended one cycle	91 (79.8%)	35 (70.0%)	
Attended both cycles	23 (20.2%)	15 (30.0%)	
**Total attendance, median (IQR)**	3 (2–6)	4 (3–9)	0.014
**Type of practice environment**			0.53
Urban	82 (71.9%)	35 (70.0%)	
Rural	27 (23.7%)	14 (28.0%)	
Not applicable	5 (4.4%)	1 (2.0%)	
**Number of patients with COVID-19 in practice**			0.97
0	45 (39.5%)	23 (46.0%)	
1–9	17 (14.9%)	7 (14.0%)	
≥10	11 (9.6%)	5 (10.0%)	
Not applicable	7 (6.1%)	2 (4.0%)	
Unknown	34 (29.8%)	13 (26.0%)	

Note: NP: nurse practitioner; MD: physician; IQR: interquartile range.

^a^ Participants who completed both pre- and post-ECHO self-efficacy questionnaire.

^b^ Will not total to 100% as participants were given the option to select more than one type of practice.

### Quantitative findings

#### Self-Efficacy

**Figure 1.**
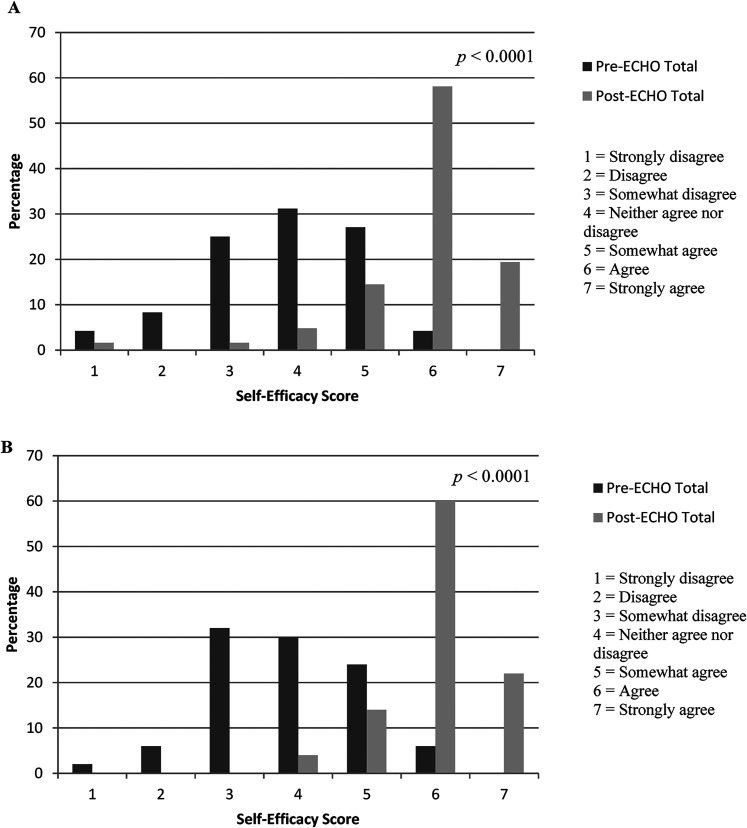
(A) percentage distribution of median pre-ECHO and post-ECHO self-efficacy scores across all 17 items for total cohort (*n* = 114), (B) percentage distribution of median pre-ECHO and post-ECHO self-efficacy scores across all 17 items for subgroup (*n* = 50).

The median self-efficacy score for the total cohort pre-ECHO was 5, ‘somewhat agree’, (IQR 4–6) and the post-ECHO score was 6 ‘agree’ (IQR 6–6) (Figure 1(A)). HCWs reported lowest self-efficacy pre-ECHO in managing their pregnant patients with COVID-19 and their patients with substance use disorder with COVID-19 (Appendix 4). In the subgroup, median scores for all items increased post-ECHO (Figure 1(B)), with the sum of all items increasing from 4.7 (IQR 4.1–5.5) pre-ECHO to 6.0 (IQR 5.5–6.4) post-ECHO (*p* < 0.0001) (Appendix 5).

There was significant improvement in self-efficacy scores for all 17 items independent of underlying participant characteristics, with the exception of male sex (*p* = 0.094) (Table 2). Similarly, ECHO participants who had been in practice for 10 or more years were found to have higher pre-ECHO self-efficacy scores compared to those with 0–9 years in practice (*p* = 0.0209), but scores increased in both groups to similar values post-ECHO.

**Table 2.** Comparison of participant characteristics and pre-post ECHO self-efficacy median scores, subgroup (*n* = 50).

	Self-efficacy score (Median, IQR)		Median difference Post versus Pre (95% CI)	*p*-value
	Pre-ECHO	Post-ECHO		
**Profession**				
NP	4.7 [4–5.4]	6 [5.3–6.4]	1.3 (0.6–1.7)	<0.0001
MD	4.8 [4.1–5.8]	6 [5.4–6.5]	1.1 (0.1–2)	0.0051
Other	4.7 [4.3–5.3]	6 [5.8–6.6]	1.4 (0.3–1.9)	0.0020
**Sex**				
Female	4.8 [4.1–5.6]	6 [5.5–6.5]	1.3 (0.8–1.5)	<0.0001
Male	4.4 [3.7–5.5]	6 [5.5–6.3]	1.6 (−0.4–4)	0.0938
**Years in practice**				
0–9	4.2 [4–5.4]^a^	6 [5.3–6.4]	1.5 (1.0–2.1)	<0.0001
≥10	4.9 [4.4–5.8]	6.1 [5.6–6.5]	0.9 (0.3–1.5)	<0.0001
**Age group**				
20–39	4.6 [4–5.5]	6 [5.3–6.4]	1.5 (0.1–2.1)	0.0013
≥40	4.8 [4.2–5.7]	6 [5.7–6.5]	1.2 (0.7–1.7)	<0.0001
**Attendance**				
Attended one cycle	4.6 [4–5.6]	6 [5.5–6.5]	1.4 (0.8–1.7)	<0.0001
Attended both cycles	4.8 [4.3–5.4]	6 [5.5–6.3]	1 (0.2–1.8)	0.0084
**Type of practice environment**				
Suburban/urban	4.5 [4–5.7]	6 [5.5–6.4]	1.3 (0.7–1.7)	<0.0001
Rural	5.1 [4.2–5.5]	6.1 [5.7–6.4]	1.1 (−0.4–2.1)	0.0134
**Number of patients with COVID-19 in practice**				
0	4.3 [4–5.5]	6 [5.3–6.3]	1.4 (0.3–1.7)	0.0007
≥1	4.8 [ 4.4–5.7]	6.2 [5.6–6.5]	1.3 (0.1–1.8)	0.0034

Note: CI: confidence interval; NP: nurse practitioner; MD: physician.

^a^ Within group difference, *p* = 0.0209.

#### Satisfaction

**Figure 2.**
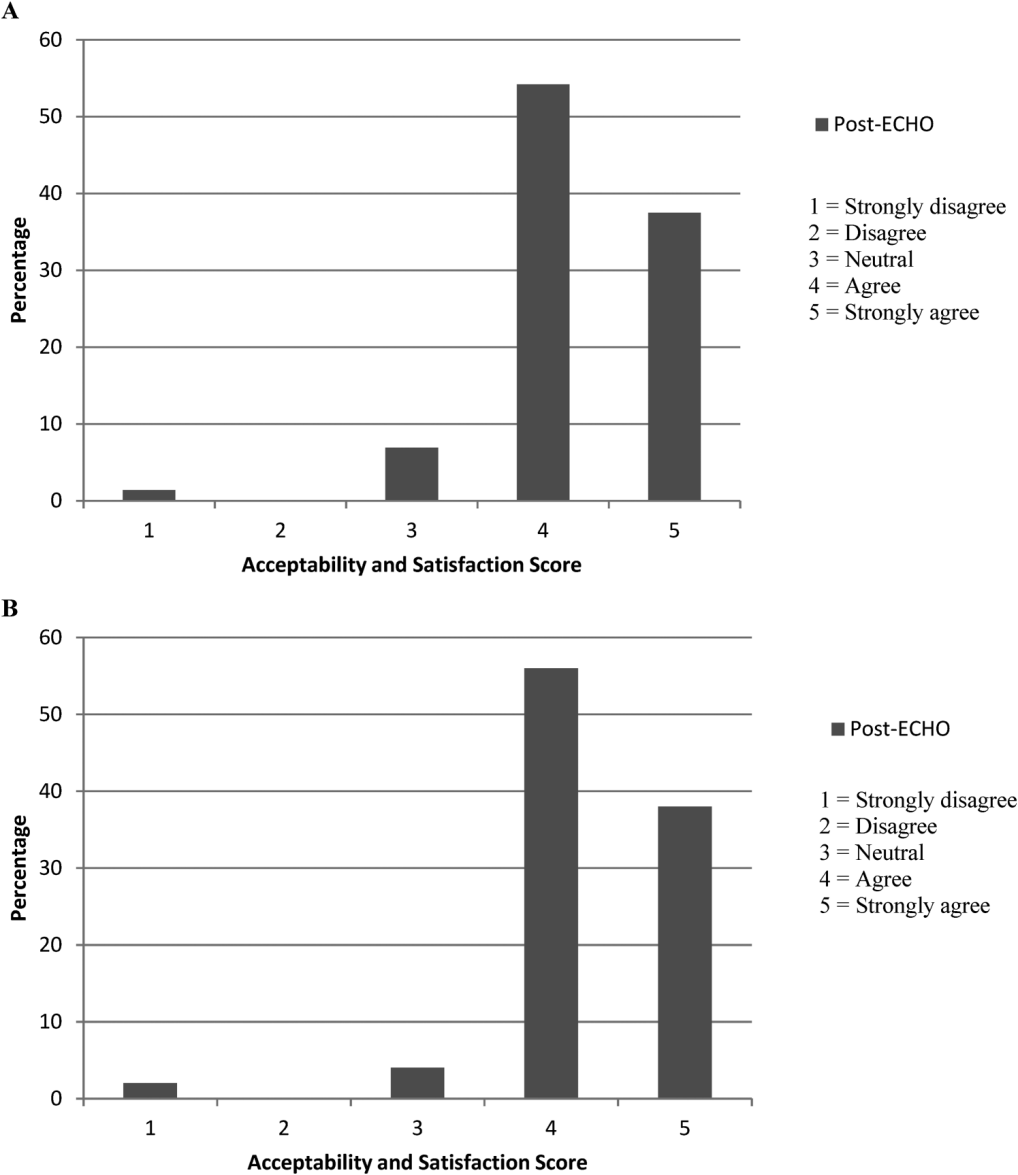
(A) percentage distribution of median acceptability and satisfaction scores across 11 items for total cohort (*n* = 114), (B) percentage distribution of median post-ECHO satisfaction scores across 11 items for subgroup (*n* = 50).

All satisfaction items were evaluated for each participant (Appendix 6). The median of all satisfaction scores for all ECHO attendees was 4 [IQR, 4–5], indicating that most participants ‘agreed’ or ‘strongly agreed’ with the majority of satisfaction items (Figure 2).

### Qualitative findings

Five participants participated in the 60-min FGD on November 24, 2020. Two were nurse practitioners, one family medicine physician, one pharmacist, and one chiropractor. They practiced at six practice settings: family health team, long-term care facility, independent private practice, aboriginal health access centre, hospital, and nurse practitioner-led clinic. Three participants (60%) practiced in urban centres in Southern Ontario, while two (40%) practiced at rural centres in Northern Ontario. Three participants (60%) identified as women, and the mean years in practice were 35.4 years (SD 11.3).

After analysing the FGD transcript, three themes were identified: (1) knowledge gained in ECHO, (2) context of practice during the COVID-19 pandemic, and (3) facilitators for ECHO program success. HCWs discussed how their unique context of practice changed during the COVID-19 pandemic (Table 3). Interestingly, virtual care increased attendance at ECHO sessions, allowing access to sessions where it may not have been feasible prior.

**Table 3.** Participant quotations supporting each theme.

Theme	Representative quotation
Knowledge gained in ECHO Asymptomatic transmission Patient experience Patient dermatological changes	Most asymptomatic transmission is pre-symptomatic and they will develop symptoms, however mild. […] There are very few asymptomatic transmissions. (P_037, NP) In terms of [the patient] and her lived experience, I think that's so powerful to hear a message like that. (P_020, Pharm) Seeing some of the dermatological changes that patients potentially experience with COVID [was helpful]. We can talk about it but when you can see it, it makes a difference. (P_028, DC)
Context of practice during the COVID-19 pandemic Different work environment: changes in clinic structure: PPE, virtual care Shut down or reduction of health care services Emotional components: burnout, stress, hopelessness Relief for no COVID-19 patients, but sense of impending Increased hospitalisation of patients with chronic diseases due to delayed care	[COVID] changed the whole dynamics and everything about the facility changed for everybody […]. I could hardly recognise some of my colleagues with all our gear, even without full PPEs we’re all in masks and shields. […] A lot of staff are extremely stressed, it's more work and the end isn't in sight. […] It's a totally different work environment to anything I have worked in, in all my [37-year] career. (P_009, MD) For myself as a chiropractor, we had a complete shut down in all the services we were providing in the province. (P_028, DC) We have been lucky because we don't have a lot of cases in the area that we cover, so for us we really dodged a bullet and I feel a nice relief. But at the same time, we have not been spurred into practice because we haven't had positive cases. (P_029, NP) We are seeing an increase in chronic diseases that are ending up in the hospital. We have 120% capacity across Northern Ontario in many of our hospitals. Our larger centres are reaching out to smaller centres to take patients that are not COVID-related and if it's not COVID-related, it's related to people being very sick because they’re not getting care early on. (P_037, NP)
Facilitators for ECHO program success Virtual event facilitated ECHO attendance Practicability and relevance of knowledge Multidisciplinary aspect of sessions Clinical pearls Perspective from the trenches Connection to community of support Connection for HCWs practicing in more isolated regions	It's actually one of the advantages of the pandemic because I’m working from home more, so can schedule around the time of the ECHO. And when I was in office more, I wouldn't have had the time to do that. (P_009, MD) There's no problem getting knowledge. The hardest part is integrating it into your practice. And that is where I find [ECHO] COVID is good. […] There is such a lag between the time the information gets out there until it's actually adopted in practice. (P_029, NP) It's also nice that the folks are multidisciplinary, and we get perspective from different people. (P_009, MD) With ECHO, I don't have to spend $500.00 [on a conference] and I get usually more than one pearl out of the whole process […] I could apply a lot of those pearls on Monday morning quite easily. (P_028, DC) A lot of the education is academic ivory tower and sometimes this gives a perspective to people in the trenches who can't be exactly like what the ivory tower is. It's made more real in that way. (P_009, MD) ECHO is a great way to connect with other specialists, […]especially in northern Ontario. It really is connecting us to the more urban centres and I also think […]it's good for others to see the work that we’re doing in northern Ontario. (P_037, NP)
Take-home points:	HCWs describe the overwhelming and shifting context of primary care medical practice during the COVID-19 pandemic in Ontario, Canada. HCWs share that they have gained knowledge from attendance at ECHO COVID telemedicine education sessions. These include knowledge around asymptomatic patient transmission, visual images of patient symptom changes, and understanding more fully a patient with COVID-19's lived experience through hospital care. Many facilitators for ECHO program success were shared: ECHO COVID offered a space for connection, community, and support amongst colleagues; ECHO offered a perspective from the trenches with other stories of frontline HCWs, and that virtual care enabled attendance at ECHO and other virtual events.

### Integration

Synthesising the quantitative and qualitative data together, the data collected illustrated a rich view of frontline HCWs during the COVID-19 pandemic in the province of Ontario. The FGD explored HCWs’ knowledge gained and offered facilitators to program success, which were not otherwise available through questionnaire data. Since knowledge acquisition through multiple choice testing methods could not be ascertained from questionnaires due to the evolving nature of COVID-19 information at the time of program implementation, data collected through the FGD described examples of knowledge change.

## Discussion

This study demonstrated that a telemedicine education program (ECHO Ontario: Managing COVID-19 Patients in the Community) had a positive impact on HCWs in primary care. Quantitative results demonstrated that median self-efficacy scores increased significantly from 5 (IQR 4–6) to 6 (IQR 6–6) (*p* < 0.0001), independent of profession and other characteristics. Participants were also highly satisfied, median score of 4 out of 5 (IQR 4–5). Qualitative results revealed that knowledge was gained through session attendance and explored the context of practice in the COVID-19 pandemic in Ontario, Canada. The participants of the FGD also shared facilitators for ECHO program success, including the transition to virtual care.

As the COVID-19 pandemic has evolved, HCWs’ learning needs have evolved as well. Findings from this study are similar to others, which demonstrate that the ECHO model is well-suited for rapid and proactive implementation, features identified that increase the strategic uptake and sustainability of telehealth interventions overall.^44–46^ Globally, 304 ECHO programs have launched in 35 countries focusing on COVID-19.^35^ Across the United States, states like Missouri, Oregon, New Mexico, and Virginia rapidly developed and launched programs in response to COVID-19.^47–52^ Response has been overwhelmingly positive, with 500 to over 9000  attendees and reports of high satisfaction. The World Health Organization partnered with the ECHO Institute to launch an ECHO program for the continent of Africa, where there was high demand for credible sources on COVID-19.^53^ Finally, in Canada, ECHO Coping with COVID (ECHO-CWC) was launched to support HCWs’ mental well-being, where they found that, again, participants were highly satisfied with initial sessions (mean = 4.26/5).^54^

The COVID-19 pandemic has been an inciting event that resulted in increased utilisation and innovation of virtual care.^5,55,56^ Virtual care has broadened access to medical care while maintaining clinical effectiveness, though success remains to be demonstrated across all clinical domains.^57–59^ Another outcome of virtual care has been increased attendance at CME events like conferences and ECHO programs. This was exemplified through this ECHO COVID program, which ran Tuesdays during clinic hours. Although attendance was high compared to other programs, findings from this study reveal that the transition to virtual care enabled participants the flexibility to attend ECHO sessions. This aligns with several studies across medical disciplines, where virtual care has enabled higher attendance at such events.^60–62^ Timing and delivery platform should be considered when planning and implementing CME events.

To our knowledge, this was one of the first studies that explored knowledge gain by HCWs in a COVID-19 telemedicine education program. Knowledge change is difficult to measure among learners, especially in CME events and with a multidisciplinary audience.^63,64^ Nonetheless, knowledge acquisition is a primary goal of education. Rather than miss this opportunity, a mixed methods study approach was used.^65,66^ For future program evaluation with an evolving clinical evidence base, qualitative research methods to measure knowledge change can be considered.

Canada is in the midst of a fourth wave: 1.58 million people have tested positive for COVID-19 with over 27,500 deaths, individuals 12 years and older are eligible to receive a vaccine, and hospital systems in some regions of Canada remain at capacity from surging acute care hospitalisations.^25,67^ This study does not capture the impact of the vaccine rollout on outcome measures. Furthermore, even though participants joined from outside of Ontario and internationally (Brazil), this data was not analysed for this study. Future research will aim to collect more qualitative data from HCWs and compare the out-of-province and international cohorts with Ontario participants. Future research will also aim to collect healthcare utilisation data through provincial administrative databases, which will investigate impact of training HCWs on clinical utilisation and costs.

As the transition begins to examining the long-term socio-economic impact of COVID, understanding new vaccines and novel therapies, and preparing for future waves, the adaptability of the ECHO COVID curricula will evolve to reflect these issues.

## Limitations

There are several limitations to this study. All questionnaires are self-reported and there was no control group for comparison of self-efficacy. A pre-post assessment, however, of self-efficacy scores within a subgroup of participants was able to be collected. There were differences in age group, years in practice, and number of patients assessed with COVID-19 between HCWs who registered but did not attend any ECHO sessions compared to HCWs who registered and attended at least one ECHO session. This can potentially be attributed to the larger number of participants who answered ‘unknown’ to the questions related to the years in practice and age group categories who attended ECHO sessions. There was also a larger observed proportion of HCWs without any COVID-19 patients in their clinical practice who registered and did not attend any ECHO sessions. Thus, the lack of perceived applicability to their practice may have accounted for the lower attendance in this group. Furthermore, qualitative data was collected only from five participants, though purposive sampling strategy was employed to ensure a representative sample participated. Given the tight time frame of this study, additional qualitative data collection was not feasible. Finally, due to the rapidly evolving knowledge base regarding COVID-19, knowledge testing using multiple choice or key feature questions was not undertaken.

## Conclusion

This study demonstrated that a telemedicine education program (ECHO Ontario: Managing COVID-19 Patients in the Community) had a positive impact on HCWs’ self-efficacy and satisfaction. The focus group discussion revealed that knowledge was gained through sessions and explored the context of practice for HCWs during the COVID-19 pandemic in Ontario. Participants of the focus group discussion also discussed facilitators for ECHO program success, including the transition to virtual care. Even though the COVID-19 pandemic has been overwhelming and pervasive, ECHO has provided an opportunity for HCWs to gain access to rapidly evolving information, best practices, and a space for community support.

## Appendix

[Table table4-1357633X211059688]

**Appendix 1. table4-1357633X211059688:** ECHO COVID curricula topic table over two cycles

Curricula topic	Cycle 1	Cycle 2
Classification of COVID-19	X	X
Pharmacotherapy for COVID-19	X	X
Respiratory health & COVID-19	X	X
Rehabilitation and pain management of patients with COVID-19	X	
Exercise for community outpatients	X	X
Palliative care & COVID-19	X	
Substance use disorders & COVID-19	X	
Treatment of long COVID	X	X
COVID-19 vaccine information and resources		X
Post-ICU syndrome after COVID-19		X
Patient mental health during the COVID-19 pandemic	X	X
Patient community resources	X	X
Special population management: patients in aggregated settings	X	
Special population management: pregnant women	X	X
Special population management: children		X

## Appendix 2. ECHO Ontario COVID-19 Focus Group Discussion Guide

Overview: The purpose behind this set of questions is to understand the changing nature of primary care in the face of the COVID-19 pandemic, the role of ECHO, and feedback for program evaluation.

- Welcome and thanks: finishing within 1 h [by 6:00 pm]
- Introduction of moderator and note-taker
- Some housekeeping for this focus group:
  - Finish by 6:00 pm
  - We are recording this session so we can transcribe this for program evaluation and research purposes later
  - No right/wrong answer; hoping that you will share your experiences regarding ECHO
  - Please don't share any patient information and please respect the confidentiality of what's said in the group.
  - If no dissent, will start with the questions

[2 min] Intro- thank you all for being here. You are all here because you’ve participated in Project ECHO: Managing COVID-19 in Primary Care. Tonight, we’re interested in exploring two main topics with you: (1) how the COVID-19 pandemic has affected your practice and work, and (2) the impact of ECHO on your clinical practice.

A. [3 min] Round of intros: name, profession, location of practice, number of years in practice
B. [15 min] We’ve all had to change the way we work in response to the COVID-19 pandemic. Responding to a rapidly changing environment, keeping up-to-date with new guidelines, and living with and in uncertainty has become a new ‘normal’ and can be overwhelming. These first set of questions we’d like to explore are around this:
  1) How has your practice changed since the pandemic started?
    Prompts: online consults, virtual assessments, PPE, staffing, patients with COVID
  2) Have you had any patients in your practice develop COVID-19?
    Prompts: management of COVID-19 patients, telemedicine consults, adhering to public health guidelines
  3) What do you foresee being the biggest challenges in your clinical practice and care in the coming months?
    Prompts: wave 2, lack of public health guidance or enforcement, burnout
C. [40 min] Thank you for that. Switching gears to ECHO now – ECHO is an education program aimed at bridging the gap between specialist and primary care. Our sessions are delivered virtually with a didactic and case presentation component.
  4) Please tell us about your experience of participating in ECHO COVID.
    Prompts: how well ECHO addressed participants’ needs, COVID compared to other ECHO programs, learning from the community
  5) Tell us about your experience of presenting a patient at ECHO.
    Prompts: how is your patient doing now, watching others present, selection process, preparing the presentation, giving the presentation.
  6) Please tell us about the ways in which ECHO has helped you navigate the pandemic.
    Prompts: COVID knowledge, fast information from knowledgeable hub members, patient navigation, transition to virtual care, dissemination to others
  7) What advantages do you get from ECHO over google or Up-to-date?
    Prompts: didactics, real patients, building community, interprofessional hub team, transitions in care (where to refer), the clinical librarian's resources provided in each session
  8) Who do you think would benefit most from ECHO?
    Prompts: patients to be presented, colleagues or other clinics to attend sessions
  9) Thinking back, can you tell us what's been the best thing for you about participating in ECHO?

We have about 5 min left. Is there anything that we haven't covered that strikes you as relevant (and brief)?

Thank you. You can also email us with any additional thoughts that occur to you after this conversation. Again, thank you so much for your time and your thoughtfulness.

## Appendix

[Table table5-1357633X211059688]

**Appendix 3. table5-1357633X211059688:** Baseline cohort characteristics between study participants (*n* =114) and registrants (*n* =125).

	Study participants^a^ (*n* = 114)	Registrants^a^ (*n* = 125)	*p*-value
**Profession**			0.0716
NP	42 (36.8%)	60 (48%)	
MD	34 (29.8%)	39 (31.2%)	
Other	38 (33.3%)	26 (20.8%)	
**Female**	99 (86.8%)	95 (76%)	0.0804
**Years in practice**			0.0009^∗^
0–9	49 (43.0%)	62 (49.6%)	
10–19	30 (26.3%)	34 (27.2%)	
≥20	21 (18.4%)	29 (23.2%)	
Unknown	14 (12.2%)	0 (0%)	
**Age group**			0.0006^∗^
20–39	47 (41.2%)	49 (39.2%)	
40–59	40 (35.1%)	64 (51.2%)	
≥60	15 (13.2%)	12 (9.6%)	
Unknown	12 (10.5%)	0 (0%)	
**Type of practice** ^b^			0.1945
Family health team	42 (36.8%)	28 (22.4%)	
Hospital	19 (16.7%)	22 (17.6%)	
Independent practice	13 (11.4%)	11 (8.8%)	
Community health center	10 (8.8%)	11 (8.8%)	
Long-term care	8 (7.0%)	3 (2.4%)	
Family health organization	6 (5.3%)	19 (15.2%)	
Other	16 (14.0%)	19 (15.2%)	
Two or more groups selected	11 (9.6%)	0 (0%)	
**Type of practice environment**			0.3062
Urban	82 (71.9%)	93 (74.4%)	
Rural	27 (23.7%)	22 (17.6%)	
Not applicable	5 (4.4%)	10 (8%)	
**Number of patients with COVID-19 in practice**			0.0023^∗^
0	45 (39.5%)	71 (56.8%)	
1–9	17 (14.9%)	25 (20%)	
≥10	11 (9.6%)	11 (8.8%)	
Not applicable	7 (6.1%)	5 (4%)	
Unknown	34 (29.8%)	13 (10.4%)	

Note: NP: nurse practitioner; MD: physician.

^a^ Study participants are HCWs who registered for ECHO COVID, attended at least one ECHO session, and completed questionnaires (the pre-ECHO questionnaire and/or the post-ECHO questionnaire). Registrants are HCWs who registered for ECHO COVID sessions but did not attend any ECHO session.

^b^ Will not total to 100% as participants were given the option to select more than one type of practice.

^∗^P < 0.05.

## Appendix

[Table table6-1357633X211059688]

**Appendix 4. table6-1357633X211059688:** Median pre-post self-efficacy scores, total cohort (*n* =114).

Item: ‘I am confident in my ability to…’				Pre-ECHO Median [IQR]	Post-ECHO Median [IQR]	Median change (95%CI)	*p*-value
1. Diagnose a patient with COVID19				5.5 [4–6]	6 [6–7]	0.5 (0–1)	0.0009
2. Identify which type of COVID-19 testing is appropriate for my patient and interpret the results				5 [3.8–6]	6 [6–7]	1 (1–1)	<0.0001
3. Identify which patients are at a higher risk for COVID-19				6 [5–7]	7 [6–7]	1 (0–1)	0.0003
4. Manage COVID-19 patients in the community who have never been hospitalised/discharged from hospital for COVID-19				5 [4–6]	6 [5.3–6.8]	1 (1–1)	<0.0001
5. Manage COVID-19 patients in the community who have been hospitalised/discharged from hospital for COVID-19				4 [3–5]	6 [5–6]	2 (1–2)	<0.0001
6. Manage my COVID-19 patients in aggregated settings (LTC, prisons, etc)				4 [3–5]	5.5 [4–6]	1.5 (1–2)	<0.0001
7. Determine when my COVID-19 patients should be admitted to hospital				5 [4–6]	6 [5–7]	1 (1–1)	<0.0001
8. Know where to access up to date resources regarding COVID-19				5 [5–6]	6 [6–7]	1 (1–1)	<0.0001
9. Address the mental health concerns of patients during the COVID-19 pandemic				5 [4–6]	6 [6–7]	1 (1–1)	<0.0001
10. Understand the presentation of COVID-19 in children				4 [3–5]	6 [5–6]	2 (1–2)	<0.0001
11. Provide care for a pregnant patient with COVID-19				3 [3–4]	5 [5–6]	2 (2–2)	<0.0001
12. Provide care for a palliative patient with COVID-19				4 [3–5]	6 [4–6]	2 (1–2)	<0.0001
13. Provide care for a patient with substance use disorder with COVID-19				3 [3–5]	5 [4–6]	2 (1–2)	<0.0001
14. Provide virtual care as appropriate for patients with COVID-19				5 [4–5]	6 [5–7]	1 (1–2)	<0.0001
15. Know how to sanitise my office to protect patients, faculty, and staff				6 [5–7]	6 [6–7]	0 (0–1)	0.0191
16. Know how to protect myself using PPE				6 [6–7]	7 [6–7]	1 (0–1)	0.0219
17. Know how to identify alternate staffing plans to ensure staff are available when needed				5 [4–6]	6 [5–7]	1 (0–1)	0.0014
*Sum of all questions*				4.7 [4.2–5.6]	6 [5.6–6.3]	1.3 (0.8–1.4)	<0.0001
Data are presented as median [interquartile range, 25–75%].							
Scoring	1	2	3	4	5	6	7
	Strongly disagree	Disagree	Somewhat disagree	Neither agree nor disagree	Somewhat agree	Agree	Strongly agree

*Not applicable* = not included.

## Appendix

[Table table7-1357633X211059688]

**Appendix 5. table7-1357633X211059688:** Median pre-post self-efficacy scores, subgroup (*n* =50).

Self-efficacy item: ‘I am confident in my ability to…’				Pre-ECHO median [IQR]	Post-ECHO median [IQR]	Median change (95%CI)	*p*-value
1. Diagnose a patient with COVID19				6 [4–6]	6 [6–7]	0 (0–1)	<0.0001
2. Identify which type of COVID-19 testing is appropriate for my patient and interpret the results				5 [4–6]	6 [6–7]	1 (1–2)	<0.0001
3. Identify which patients are at a higher risk for COVID-19				6 [5–6.3]	7 [6–7]	0 (0–1)	<0.0001
4. Manage COVID-19 patients in the community who have never been hospitalised/discharged from hospital for COVID-19				5 [4–6]	6 [6–7]	1 (1–2)	<0.0001
5. Manage COVID-19 patients in the community who have been hospitalised/discharged from hospital for COVID-19				5 [3–5.3]	6 [5–6]	1 (0–2)	<0.0001
6. Manage my COVID-19 patients in aggregated settings (LTC, prisons, etc)				4 [3–5]	6 [4–6]	2 (1–2)	<0.0001
7. Determine when my COVID-19 patients should be admitted to hospital				5 [4–6]	6 [6–7]	1 (0–1)	<0.0001
8. Know where to access up to date resources regarding COVID-19				6 [5–6]	6 [6–7]	1 (0–1)	0.0002
9. Address the mental health concerns of patients during the COVID-19 pandemic				5 [4–6]	6 [6–6.8]	1 (1–2)	<0.0001
10. Understand the presentation of COVID-19 in children				4 [3–5]	6 [5–6]	2 (1–2)	<0.0001
11. Provide care for a pregnant patient with COVID-19				3 [3–4]	6 [5–6]	2 (1–3)	<0.0001
12. Provide care for a palliative patient with COVID-19				4 [3–5]	6 [5–6.3]	2 (1–2)	<0.0001
13. Provide care for a patient with substance use disorder with COVID-19				4 [3–5]	5 [4–6]	1 (1–2)	0.0001
14. Provide virtual care as appropriate for patients with COVID-19				5 [3.8–5]	6 [6–7]	2 (1–2)	<0.0001
15. Know how to sanitise my office to protect patients, faculty, and staff				6 [5–7]	6 [6–7]	1 (0–1)	0.0006
16. Know how to protect myself using PPE				6 [6–7]	6 [6–7]	0 (0–1)	0.0316
17. Know how to identify alternate staffing plans to ensure staff are available when needed				5 [4–6]	6 [5–7]	1 (0–1)	0.0023
*Sum of all questions*				4.7 [4.1–5.5]	6 [5.5–6.4]	1.3 (0.8–1.7)	<0.0001
Data are presented as median [interquartile range, 25–75%].							
Scoring	1	2	3	4	5	6	7
	Strongly disagree	Disagree	Somewhat disagree	Neither agree nor disagree	Somewhat agree	Agree	Strongly agree

*Not applicable* = not included.

## Appendix

[Table table8-1357633X211059688]

**Appendix 6. table8-1357633X211059688:** Median satisfaction scores, total cohort (*n* =114).

Item					Satisfaction scores Median [IQR]
1) Involvement in project ECHO was a worthwhile experience for me					5 [4–5]
2) I will recommend involvement in project ECHO to my colleagues					5 [4–5]
3) I believe that the quality of care I provide my patient has improved considerably as a result of my involvement in project ECHO					4 [4–5]
4) The goals and objectives I had upon becoming involved in project ECHO have been met					4 [4–5]
5) My participation in project ECHO has enhanced my professional satisfaction					4 [4–5]
6) Project ECHO has diminished my professional isolation					4 [4–5]
7) Collaboration among agencies in project ECHO is a benefit to my clinic					4 [3–5]
8) Project ECHO has expanded its access to effective COVID-19 treatment for patients in our community					4 [3–5]
9) Project ECHO has improved quality and safety of patient care					4 [4–5]
10) Project ECHO has enabled rapid learning and best practice dissemination					4 [4–5]
11) Project ECHO has reduced variations in care					4 [4–5]
*Sum of all questions*					4.2 [3.9–4.8]
Data are presented as median [interquartile range, 25–75%].					
Scoring	1	2	3	4	5
	Strongly disagree	Disagree	Neutral	Agree	Strongly agree

*Not Applicable* = Excluded.
